# Transforming a Large-Enrollment Neuroscience Course With Active Learning: Using AI-Based Text Analysis to Capture What Traditional Evaluations Miss

**DOI:** 10.59390/001c.163545

**Published:** 2026-06-17

**Authors:** Mariana Teles, Erin Clabough, Taylor Byron

**Affiliations:** 1 Psychology University of Virginia

**Keywords:** active learning, student feedback analysis, large enrollment courses

## Abstract

Large-enrollment undergraduate neuroscience courses often rely on traditional lecture formats that limit student engagement with complex material. This study describes the redesign of a large-enrollment neuroscience course into a hybrid active learning format combining asynchronous online lectures with weekly small-group in-person sessions. To evaluate the impact of this redesign, we analyzed 547 open-ended student comments from 232 students across two semesters (one traditional lecture, one active learning) using zero-shot classification (an approach that uses a pre-trained language model to categorize text into researcher-defined themes), via an open-source large language model (BART), and compared these findings with Likert-scale questions. The active learning format produced significant improvements in students’ perceived learning experience and engagement, gains that were not visible in the Likert-scale data. This discrepancy highlights the limitations of Likert-scale evaluation tools for capturing the nuanced benefits of pedagogical innovations in large classrooms. We provide a practical framework neuroscience educators can adopt to redesign large courses and assess their impact using accessible AI tools, without requiring specialized technical expertise.

## Introduction

Large-enrollment introductory neuroscience courses (typically 100 or more students) face a persistent challenge: how do you keep students actively engaged with complex, abstract material such as synaptic transmission, neural circuits, and cognitive systems, when hundreds of students sit in a lecture hall designed for passive reception? Traditional lecture formats, while efficient for content delivery, offer limited opportunities for the kind of effortful processing and active retrieval, such as practice testing, spaced review, and elaborative questioning. These are strategies that cognitive science research consistently links to durable learning (Freeman et al., 2014; Prince, 2004). The need to develop effective active learning alternatives is especially pressing in these foundational courses, which often serve as entry points for students considering neuroscience majors and careers.

Traditional assessment methods, such as end-of-semester evaluations with Likert-scale questions, have long been the standard for gauging teaching effectiveness and student satisfaction. However, these tools often fail to capture the nuanced aspects of student experiences, particularly when evaluating novel pedagogical approaches (Uttl et al., 2017). The limitations of these conventional metrics become even more pronounced in the context of active learning implementations, where the multifaceted nature of student engagement and learning may not be adequately reflected in numerical ratings (Carr et al., 2015).

Moreover, while established qualitative research traditions, including thematic analysis (Braun & Clarke, 2006), grounded theory, and content analysis, offer rigorous frameworks for interpreting open-ended feedback, these approaches are time-intensive and require substantial methodological training. When applied to large-enrollment courses generating hundreds of comments per semester, manual qualitative coding becomes impractical for most instructors, even when the methods themselves are well-validated (Alhija & Fresko, 2009).

The advent of advanced Natural Language Processing (NLP) techniques, particularly those leveraging Large Language Models (LLMs), offers a promising solution to these assessment challenges, especially the challenge of analyzing large volumes of qualitative feedback. These technologies can analyze vast amounts of textual data efficiently, extracting meaningful patterns and insights that might be overlooked in manual analysis (Rodríguez-Ruiz et al., 2021). A growing body of work has begun to explore the use of LLMs for qualitative data analysis in educational and social science research, including LLM-assisted content analysis for coding large volumes of student feedback and research texts (Chew et al., 2023; Gale & Nicolajsen, 2025) and deductive coding of open-ended survey responses (McClure et al., 2024). However, applications of these methods to the assessment of active learning interventions in particular remain limited.

To bridge this research gap, the current study uses LLMs to examine the impact of incorporating active learning components into a large-enrollment undergraduate neuroscience course. We employ an innovative approach using zero-shot classification, which is a technique in which a pre-trained language model categorizes text into researcher-defined themes without requiring labeled training data. This was implemented via Meta’s BART to analyze open-ended student feedback. This method allows for a nuanced, multi-dimensional assessment of student experiences without the time-consuming manual coding or potential researcher bias associated with traditional qualitative analysis. This method may also offer a flexible and scalable approach to feedback analysis, which could be utilized in a variety of educational contexts.

By comparing the results of the LLM analysis with traditional course evaluation metrics, this study seeks to provide a more comprehensive understanding of the strengths and limitations of different assessment methods for capturing the impact of pedagogical innovations, such as the implementation of active learning components. This approach not only offers insights into the effectiveness of active learning strategies in large enrollment courses but also demonstrates the potential of advanced LLM techniques to transform how we analyze and interpret student feedback.

## Background

### Active Learning in Large-Enrollment Neuroscience Courses

Active learning is defined as instructional approaches that require students to engage in meaningful activities and think about what they are doing, rather than passively receiving information (Prince, 2004). Research testing active learning approaches in the biological and brain sciences provides strong support for moving beyond passive lecture formats (Clabough, 2025; Freeman et al., 2014; Lombardi et al., 2021).

Studies in introductory biology and neuroscience courses have documented improvements in conceptual understanding, course retention, and scientific reasoning when instructors incorporate structured active learning elements (Freeman et al., 2014; Lombardi et al., 2021). However, large-enrollment courses, often the primary gateway into neuroscience programs, present particular logistical challenges for active learning implementation, including limited physical space, high student-to-instructor ratios, and heterogeneous student preparation. Active learning approaches such as flipped classrooms, peer instruction, and structured small-group activities have been tested in these contexts with promising but variable results, underscoring the importance of rigorous, context-sensitive assessment of their outcomes (Deslauriers et al., 2019; Knudson & Wallace, 2021; Poirier & Feldman, 2007).

In particular, Clabough (2025) introduced the Knowledge Flow framework, a neuroscience-informed model that organizes active learning strategies into seven interconnected “Knowledge Catalysts” (e.g., scaffolding, belonging, collaboration) designed to create the conditions for deep learning in large courses. Implemented in a 300-student introductory neuroscience course using a hybrid format combining asynchronous lectures with weekly small-group sessions, the Knowledge Flow approach produced significant improvements in exam performance compared to traditional lecture formats. The course redesign examined in the present study was developed within this framework.

To understand how current assessment limitations affect our ability to evaluate active learning effectiveness, it is essential to examine what research reveals about active learning’s complex benefits and implementation challenges. Active learning’s documented benefits across disciplines include enhanced student engagement, critical thinking development, and improved academic performance (Prince, 2004). These multifaceted outcomes, while highly aligned with educational goals, often require qualitative assessment to fully capture the complex nature of student learning experiences, an aspect that traditional evaluation methods struggle to address.

Researchers have also explored the impact of active learning on specific cognitive processes. Cherney (2008) examined how this approach influences students’ memory retention, using pre- and post-tests alongside surveys to capture both performance improvements and student perceptions. The author concluded that active learning not only enhances immediate recall but also fosters long-term retention, providing valuable insights into the cognitive benefits of these strategies. These cognitive benefits, while demonstrable through controlled pre- and post-testing, may be difficult to detect through traditional end-of-semester course evaluations that rely primarily on Likert-scale ratings.

However, implementing active learning techniques presents unique challenges, particularly in large enrollment courses. The scale of these classes can make it difficult to facilitate meaningful interactions and provide personalized feedback. To address these challenges, educators have developed a range of innovative strategies, including pop-up quizzes, structured group discussions, and small group challenges (Deslauriers et al., 2019; Schmidt & Libre, 2020). These approaches aim to break down the anonymity of large classes and create opportunities for active participation.

Several studies illustrate strategies for implementing and assessing active learning at scale. Poirier and Feldman (2007) demonstrated that Individual Response Technology in large introductory psychology courses increased exam performance and was positively received by students, while Knudson and Wallace (2021) found that low-tech active learning exercises in biomechanics courses produced meaningful learning gains without expensive technology. However, both studies relied on traditional Likert-style survey methods to capture student perceptions, which may not fully reveal nuanced aspects of students’ experiences. This ongoing tension with effective implementation paired with limited assessment tools highlights the need for more sophisticated analytical methods that can extract deeper insights from student feedback.

### Assessing the Impact of Active Learning Pedagogies

Despite the growing adoption of active learning strategies, assessing the impact of innovative approaches to engage learners remains a complex task, particularly in large classes that contain hundreds of students. Traditional methods of evaluation, such as end-of-semester student surveys, often provide limited insights into the nuanced effects of active learning interventions. Moreover, analyzing open-ended student feedback in these evaluations can be time-consuming and difficult to systematize at scale (Alhija & Fresko, 2009).

As evidence for active learning’s effectiveness has accumulated, assessment methods have evolved from simple comparisons of exam scores to more nuanced approaches that consider multiple dimensions of student engagement, perceived learning, and learning outcomes. Meta-analyses and large-scale studies have provided strong evidence for the efficacy of active learning, with one of the most influential being the comprehensive review conducted by Freeman et al. (2014). This study analyzed 225 studies comparing student performance in undergraduate STEM courses under traditional lecturing versus active learning conditions. The study found that active learning increased performance by 0.47 standard deviations on average and reduced failure rates by a factor of 1.5, with particularly strong effects on conceptual understanding. These benefits were observed across class sizes, though they appeared largest in smaller courses. This finding is particularly relevant for large enrollment courses, where the challenge lies in both implementing active learning effectively and accurately assessing its impact on deeper learning outcomes.

Building on this quantitative approach, researchers have increasingly incorporated open-ended questions about student perceptions and experiences into their assessments. For instance, Smith and Cardaciotto (2011) investigated the effectiveness of active learning in large lecture-based classes, finding that while active learning exercises led to greater retention and engagement, they did not necessarily increase student enjoyment. This finding highlights the importance of considering both cognitive and affective dimensions when evaluating active learning strategies and demonstrates why traditional satisfaction-based evaluations may not capture the full picture of active learning’s impact.

### Assessing Teaching Innovations: Limitations of Traditional Evaluations and the Promise of AI-Based Tools

Despite the value of active learning redesigns, measuring their impact on student experience remains methodologically challenging, particularly when relying on standard course evaluation instruments. End-of-semester Likert-scale surveys, the most common form of teaching feedback in higher education, offer a limited window into the student experience. Their numerical format collapses the multidimensional nature of active learning, which includes engagement, perceived learning, enthusiasm, and sense of belonging, into a small set of aggregate ratings that may obscure meaningful variation across these dimensions (Carr et al., 2015).

These limitations are compounded by well-documented validity concerns with standard course evaluation instruments. Uttl et al.’s (2017) meta-analysis of 97 studies found that SET ratings showed effectively zero correlation with student learning outcomes once prior knowledge was controlled for, and that ratings were more strongly associated with grade expectations than with learning gains. These validity concerns apply to course evaluation instruments broadly and are not resolved simply by shifting to open-ended formats, which carry their own limitations including potential response biases (Alhija & Fresko, 2009). Rather, the argument for supplementing Likert-scale items with open-ended analysis is one of dimensionality: open-ended responses can capture aspects of student experience, such as the perceived value of specific course structures, that fixed-scale items are not designed to measure. Together, these issues make Likert-scale instruments particularly ill-suited for detecting the kinds of experiential improvements that active learning interventions are designed to produce.

These limitations of both Likert-scale items and manual qualitative coding are especially problematic for active learning environments, where students may initially find new formats demanding or unfamiliar, potentially depressing satisfaction ratings even when learning is occurring. Open-ended evaluation comments offer richer information but introduce their own challenges: manual coding is time-intensive, prone to researcher bias, and impractical at the scale of large-enrollment courses where hundreds of comments may be generated in a single semester (Alhija & Fresko, 2009). As a result, instructors often either skim open-ended responses superficially or forgo systematic analysis altogether, leaving potentially valuable feedback unused.

Zero-shot classification via models such as BART offers a practical solution, enabling faculty to classify hundreds of open-ended comments in minutes on a standard laptop without specialized programming expertise (Yin et al., 2019). A detailed methodological guide, including annotated R code and validation procedures, is available in Teles and Tomasevic (2026). The present paper focuses on what this method reveals about the impact of an active learning redesign in a large-enrollment neuroscience course, and what neuroscience educators can learn from implementing both the redesign and the assessment approach.

## The Current Study

Recent trends in higher education pedagogy have emphasized the importance of active learning strategies to enhance student engagement and perceived learning. In response, a redesign of a large-enrollment neuroscience course was implemented by its instructor at a large public research university to incorporate active learning pedagogy. The initiative aimed to transform traditional lecture-based courses into more interactive and engaging learning experiences, addressing the challenges often associated with teaching introductory-level content to large student cohorts while also providing an opportunity to evaluate innovative assessment approaches for capturing the impact of these pedagogical changes.

Previously, this 2000-level (sophomore-level) neuroscience course, Survey of Neural Basis of Behavior, enrolled approximately 200 students and was delivered in a traditional lecture format. Students attended classes in large auditoriums, where their opportunities for active participation were limited to asking questions or making comments addressed to the instructor. While this format allowed for efficient delivery of content to a large number of students, it often resulted in a passive learning experience. The instructor recognized that merely listening to lectures does not necessarily equate to effective learning, especially when dealing with complex theoretical issues that might seem distant from students’ experiences and reality.

The redesigned course adopted an innovative hybrid structure, guided by the Knowledge Flow framework (Clabough, 2025), that combines online asynchronous lectures with in-person active learning sessions. First, the core content is delivered through online lectures that students can access at their own pace, allowing for flexible learning schedules while ensuring comprehensive coverage of theoretical material. Second, students attend smaller, more intimate classroom settings once a week for synchronous interactive activities. These sessions are designed to reinforce and apply the concepts learned in the online lectures, rather than introducing substantial new information. The active learning component utilizes a 99-person classroom with 11 tables of 9 students each, allowing for collaborative group work and more personalized instruction. Third, during the active learning sessions, students engage in discussions and hands-on activities that guide the connection of theoretical concepts to practical applications through interdisciplinary approaches. Finally, both graduate and undergraduate teaching assistants play a crucial role in facilitating the active learning sessions and providing real-time feedback to students.

The active learning sessions were designed around cognitive science principles known to enhance retention and transfer, including retrieval practice, elaborative interrogation, and spaced repetition (Roediger & Butler, 2011), and structured using the Knowledge Catalysts from the Knowledge Flow framework (Clabough, 2025), a neuroscience-informed model that organizes active learning strategies into seven interconnected “Knowledge Catalysts” (e.g., scaffolding, belonging, collaboration) designed to create the conditions for deep learning in large courses. Each Catalyst is grounded in a neuroscience principle. For example, the scaffolding catalyst draws on the concept of scaffold proteins that support neural growth, and the collaboration catalyst connects to cortical column organization.

In-person sessions included activities such as case-based analysis of neurological conditions, diagram reconstruction tasks (e.g., drawing and labeling neural circuits from memory), and small-group debate on topics such as the validity of localization-of-function claims.

The purpose of the current study was to examine the impact of incorporating active learning components into a large-enrollment neuroscience course and to demonstrate the utility of advanced NLP techniques in analyzing student feedback. We aimed to provide a comprehensive assessment by comparing the insights gained from NLP analysis of open-ended student responses with traditional course evaluation metrics. Specifically, we addressed the following research questions:

1. How does the integration of active learning components into large-enrollment neuroscience course affect students’ perceived learning, engagement, and enthusiasm for course content, compared to a traditional lecture format?
2. Do AI-based text analysis tools (specifically zero-shot classification using an open-source LLM) reveal dimensions of student experience that are not captured by standard Likert-scale course evaluations?
3. What practical lessons from this course redesign can guide neuroscience educators seeking to implement active learning in large-enrollment courses?

## Method

### Data Collection

The data for this study were collected from course evaluations of a 2000-level undergraduate neuroscience course, Survey of Neural Basis of Behavior (approximate enrollment: 200 students per semester), taught to undergraduate students in the College of Arts and Sciences at a large public research university. These evaluations were standard questionnaires given to students in all courses administered by the University. The data analyzed here are from the neuroscience course only and are distinct from the dataset reported in Teles and Tomasevic (2026), which applied the same analytical method to an Introduction to Cognition course. The study focused on two types of data: responses to open-ended questions and responses to Likert-scale questions.

#### Open-ended Questions

Responses to four open-ended questions from the course evaluations were analyzed:

1. “Please tell us briefly how any of the above learning activities (or other activities not included above) contributed to your learning in this course.”
2. “What would you like the instructor and university administrators to know about your experience in this course?”
3. “Please give specific examples as to how the instructor created an environment that respected difference and welcomed diverse perspectives.”
4. “What constructive suggestions do you have to help the instructor to improve this course for future students?”

#### Likert-scale Questions

Responses to two Likert-scale questions from the course evaluations were also included in the analyses. These questions were rated on a 5-point scale, with higher scores indicating stronger agreement:

1. “The course increased my enthusiasm for the topic.”
2. “Through this course, I gained a deeper understanding of the subject matter.”

Data were collected from two semesters: one semester in which the course was taught in a traditional lecture format (Control) and another implementing an active learning format (Active Learning). The control semester included 119 completed evaluations, and the active learning semester included 113 completed evaluations.

The students’ responses were categorized into two groups: Control (traditional lecture) and Active Learning. Individual-level responses to the Likert-scale items were not available from the institutional evaluation system; only aggregate means are reported. Consequently, inferential tests comparing formats on these items could not be conducted. We therefore describe the Likert-scale results as showing minimal differences rather than claiming equivalence. Direct knowledge-based outcome measures, such as course grades or exam scores, were not included in this analysis. The transition from the traditional lecture format to the active learning format involved changes not only to instructional delivery but also to the assignment structure and assessment design. As a result, grades and exam scores across the two semesters are not directly comparable and could not serve as a valid measure of the effect of the instructional format itself.

### Data Analysis

#### LLM Analysis of Open-ended Responses

Open-ended responses were analyzed using zero-shot classification, a natural language processing technique that allows a pre-trained language model to assign text to categories defined by the researcher without requiring manually labeled training data. For this study, we defined four categories grounded in themes identified inductively from students’ open-ended comments and aligned with dimensions of student experience central to active learning: Better Learning Experience, Learned More, Engaged More, and More Excited About the Content. These categories were selected to capture perceived experience across cognitive, behavioral, and affective dimensions, rather than direct measures of learning outcomes. Full definitions and the rationale for each category are provided below.

This approach leverages the model’s general language understanding to estimate how well a given text aligns with each category label, producing a probability score for each category. We used Meta’s BART (Bidirectional and Auto-Regressive Transformers; Lewis et al., 2020), a large language model designed for natural language understanding tasks, implemented through the *transforEmotion* package in R (Tomasevic et al., 2022).

BART was selected for three reasons: it is open-source and freely available, it runs locally on a standard laptop without requiring cloud computing or API access, and it has demonstrated strong performance on zero-shot classification benchmarks (Yin et al., 2019). These characteristics make it accessible to instructors without specialized computational resources or machine learning expertise.

We defined four categories reflecting dimensions of student experience relevant to active learning assessment. These categories represent themes identified in students’ open-ended comments and reflect perceived experience rather than direct measures of learning outcomes: Better Learning Experience (comments describing course structure and instructional design as facilitating learning), Learned More (comments expressing perceived knowledge and skill acquisition), Engaged More (comments reflecting active participation and involvement), and More Excited About the Content (comments conveying interest and enthusiasm toward the subject). The category labels are shorthand descriptors for the textual themes they capture; for example, “Learned More” indicates that students described themselves as having learned more, not that objective learning gains were measured.

Each student’s responses across all four open-ended prompts were concatenated into a single text string and scored against these four categories. The model produces a probability score (0–1) for each category, representing the degree to which the student’s combined response aligns with that theme. These probability scores were then standardized to Z-scores to place all categories on a common scale, facilitating comparison across teaching formats. Z-score transformation was used because raw probability scores can differ in their distributional properties across categories; standardizing ensures that differences between groups reflect relative shifts in each category rather than artifacts of differing baseline probability distributions. The complete workflow, including preprocessing steps, code, and validation procedures, is available in Teles and Tomasevic (2026).

#### Analysis of Likert-scale Responses and Comparison of NLP

The aggregate mean scores for the two Likert-scale questions, as provided by the institutional evaluation system, were compared descriptively across teaching formats (Control vs. Active Learning). Because only aggregate Likert-scale means were available, a direct statistical comparison between the NLP classification scores and the Likert-scale responses was not possible. Instead, we compared the two approaches descriptively: the NLP results were examined for differences between teaching formats, and these patterns were contrasted with the aggregate Likert-scale means for the same courses. Table 1 maps each NLP category to its definition and the Likert-scale item capturing the most conceptually similar dimension.

**Table 1.** NLP Categories and Corresponding Likert-Scale Item

**NLP Category**	**Definition**	**Corresponding Likert-Scale Item**
Better Learning Experience	Comments describing course structure and instructional design as facilitating learning	“Through this course I gained a deeper understanding of the subject matter”
Learned More	Comments expressing perceived knowledge and skill acquisition	“Through this course I gained a deeper understanding of the subject matter”
Engaged More	Comments reflecting active participation and involvement	“The course increased my enthusiasm for the topic”
More Excited About the Content	Comments conveying interest and enthusiasm toward the subject	“The course increased my enthusiasm for the topic”

## Ethical Consideration

This study utilized anonymized course evaluation data collected though standard university procedures. The study was approved by the University of Virginia Institutional Review Board for the Social and Behavioral Sciences (Protocol #7653). All data were anonymized prior to analysis, and no individual students can be identified from the results. The course evaluation system does not provide instructors with access to student names or identifying information; responses are anonymous by design. Regarding the use of NLP methods, the zero-shot classification approach categorizes text into broad thematic categories (e.g., engagement, learning experience) rather than extracting individually identifying content. Additionally, all analyses were conducted locally using open-source software, meaning student responses were not transmitted to external servers or third-party APIs. Nonetheless, we recognize that applying computational text analysis to student feedback raises broader considerations about transparency and the potential for algorithmic misclassification, and we encourage institutions adopting these methods to communicate clearly with students about how their open-ended responses may be analyzed.

## Results

**Figure 1.**
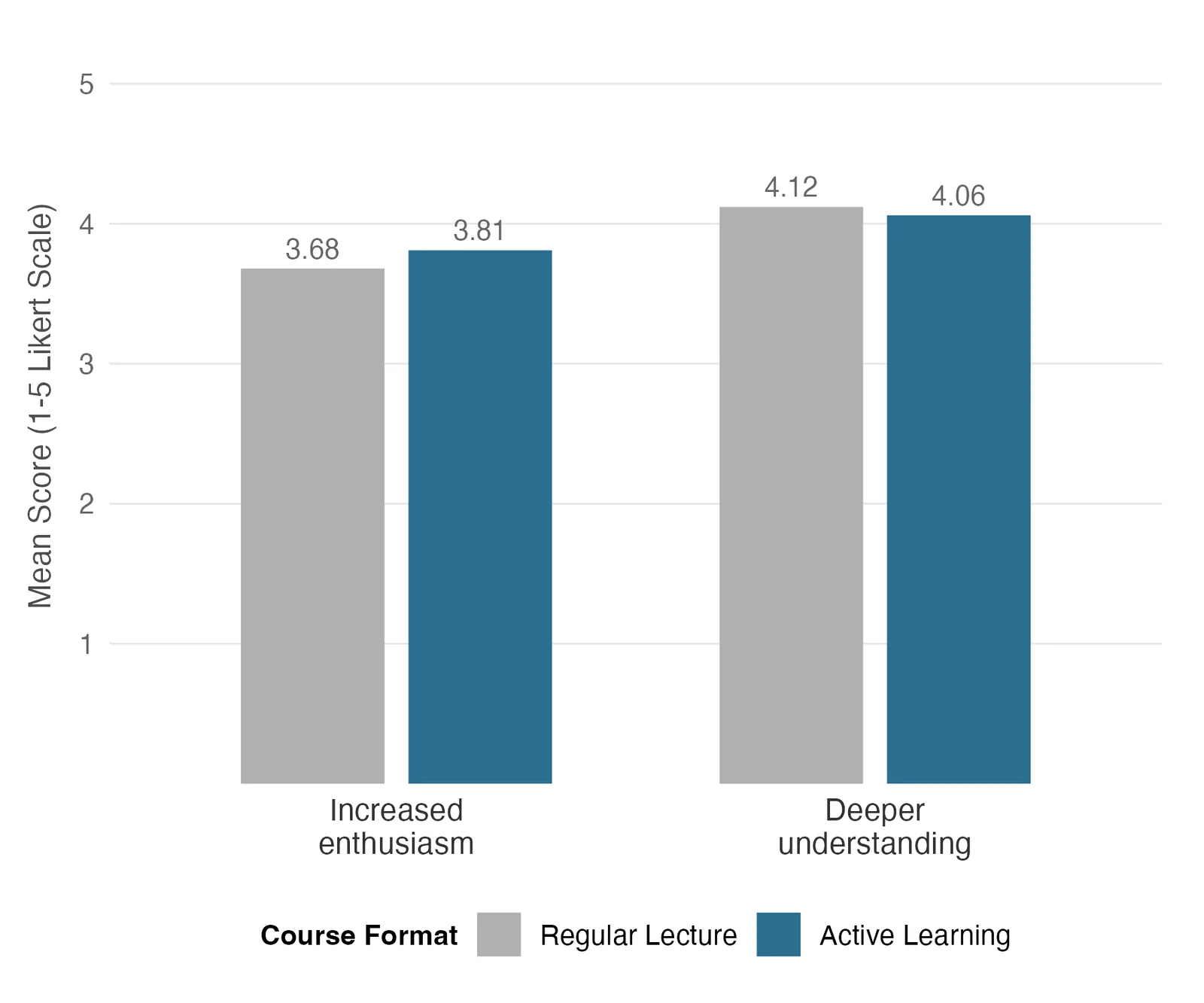
Comparison of likert-scale responses from control (n = 119) and active learning (n = 113) evaluations. “Increased Enthusiasm” represents the question “The course increased my enthusiasm for the topic.” “Deeper Understanding” represents the question “Through this course I gained a deeper understanding of the subject matter.” Y-axis values represent mean scores on a 5-point Likert scale. Individual-level responses were not available for this course, so no variability estimates are displayed.

The comparison of the Likert-scale responses showed small or no differences between the control (regular lecture) and the active learning format, as portrayed in Figure 1. Only aggregate means were available from the institutional evaluation system; individual-level responses were not provided, precluding computation of variability estimates or inferential tests. We therefore describe the Likert-scale results descriptively rather than claiming statistical equivalence between formats.

### LLM Analysis Results

**Figure 2.**
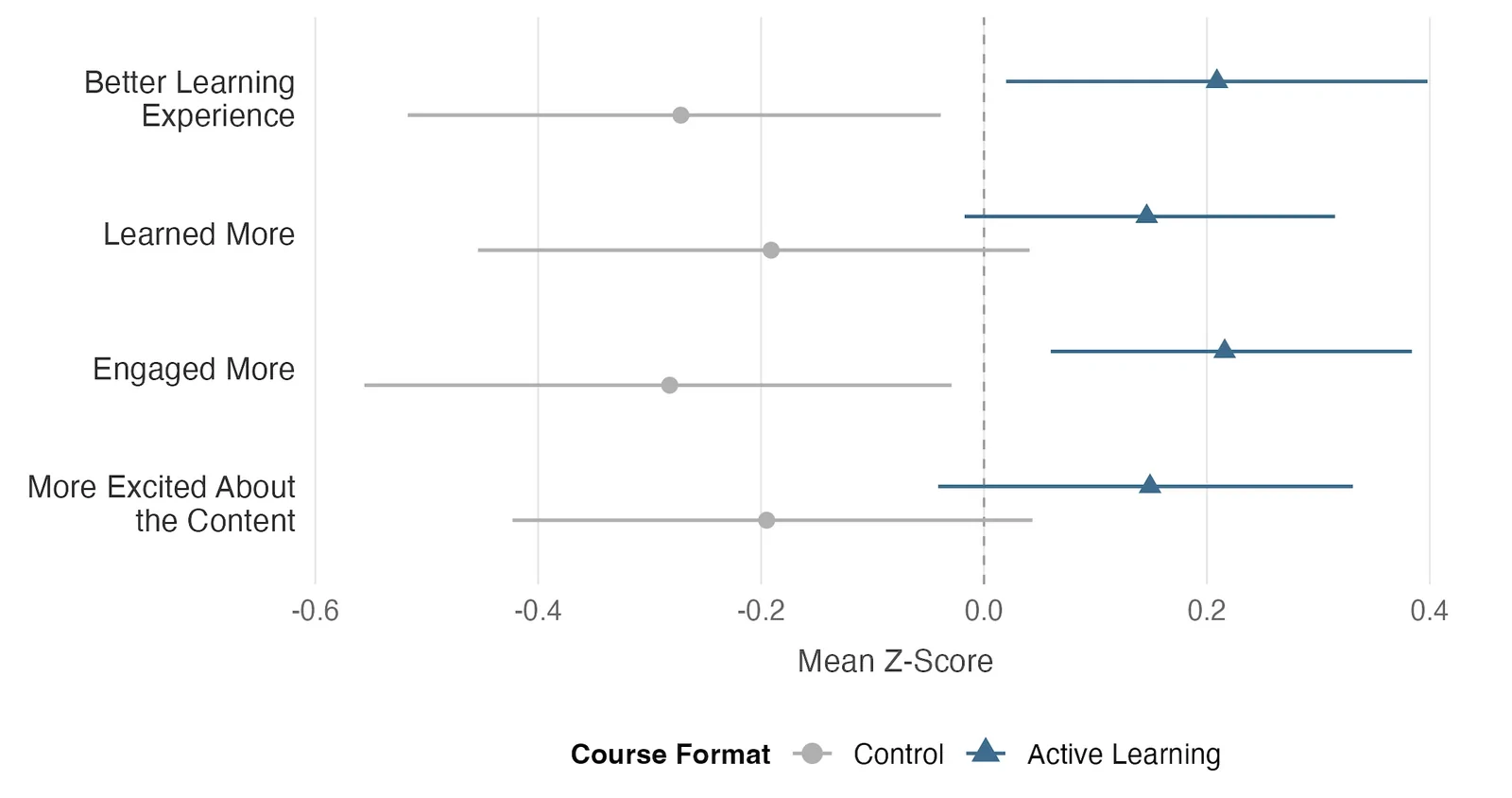
Comparison of student perceptions between control (n = 119) and active learning (n= 113). Error bars represent 95% confidence intervals for the mean Z-scores.

Individual-level validation of this classification approach was not possible for the present dataset as individual Likert-scale responses were not available; however, the same method and categories were validated in a separate course with positive evidence of discriminant validity (see Teles & Tomasevic, 2026). The current analysis compared the Control semester with the Active Learning semester across four key categories as shown in Figure 2 and Table 2.

**Table 2.** Comparison of LLM classification scores between active learning and control formats

Category	Active Learning M (95% CI)	Control M (95% CI)	Cohen's d
Better Learning Experience	0.21 (0.02, 0.40)	−0.27 (−0.52, −0.04)	0.41
Engaged More	0.22 (0.06, 0.38)	−0.28 (−0.56, −0.03)	0.42
Learned More	0.15 (−0.02, 0.32)	−0.19 (−0.45, 0.04)	0.30
More Excited About the Content	0.15 (−0.04, 0.33)	−0.20 (−0.42, 0.04)	0.29

*Note.* Survey of Neural Basis of Behavior course. Active Learning n = 113; Control n = 119. Wilcoxon rank-sum tests. Exact p-values and Bonferroni-corrected significance tests are reported in the text.

For the Better Learning Experience category, students in the Active Learning format reported higher satisfaction (M = 0.21) compared to those in the Control format (M = −0.27). The Wilcoxon rank-sum test revealed a significant difference (p = .0034, Bonferroni-adjusted α = .0125) with a small-to-medium effect size (Cohen’s d = 0.41), indicating a meaningful improvement in perceived learning experience. For Engagement, the Active Learning format resulted in higher levels of engagement (M = 0.22) compared to the Control format (M = −0.28). This difference was also statistically significant after correction (p = .005, Bonferroni-adjusted α = .0125), with a small-to-medium effect size (Cohen’s d = 0.42).

Regarding perceived learning, students in the Active Learning format reported learning more (M = 0.15) than those in the Control format (M = −0.19). However, this difference was not statistically significant (p = .068, Cohen’s d = 0.30). Similarly, in terms of Content Excitement, students in the Active Learning format showed slightly higher enthusiasm (M = 0.15) compared to the Control format (M = −0.20). This difference was also not statistically significant (p = .059, Cohen’s d = 0.29). While neither Learned More nor Content Excitement reached statistical significance, both showed trends in the expected direction.

### Comparison of NLP and Likert-scale Results

Interestingly, the NLP analysis of open-ended responses and the Likert-scale questions yielded some contrasting results. While the NLP analysis showed significant improvements in learning experience and engagement in the Active Learning format, with positive but non-significant trends in perceived learning and content excitement, the Likert-scale responses showed virtually no differences between formats. These discrepancies suggest that the NLP analysis may be capturing aspects of student experience that are not fully reflected in the more direct Likert-scale questions. The open-ended responses analyzed through NLP may provide a more comprehensive picture of students’ perceptions and experiences with the Active Learning format.

It is worth noting that the coarseness of the Likert-scale data, which is limited to aggregate means without individual-level responses, illustrates a key limitation of standard institutional evaluation systems. While these instruments yielded two summary numbers per question with no distributional information, the AI-based analysis of open-ended comments from the same students produced detailed, testable comparisons across multiple dimensions of student experience.

## Discussion

The NLP findings consistently demonstrated that the implementation of active learning strategies in the large-enrollment neuroscience course led to significant improvements in students’ perceived learning experience and engagement, with positive trends in perceived knowledge acquisition and content enthusiasm. These results align with and extend a broad base of research on the benefits of active learning in higher education (Freeman et al., 2014; Lombardi et al., 2021; Prince, 2004). Students reported a significantly better learning experience in the active learning format compared to the traditional lecture format. This finding is consistent with multiple lines of evidence showing that active learning enhances student engagement and perceived learning in large courses (Cherney, 2008; Deslauriers et al., 2019; Poirier & Feldman, 2007; Smith & Cardaciotto, 2011), and extends those findings to a neuroscience-specific context assessed through AI-based analysis of open-ended feedback.

The significant increase in student engagement observed also corroborates the work of Poirier and Feldman (2007), who demonstrated that the use of Individual Response Technology (IRT) (i.e., clickers) in large introductory psychology classes increased student engagement and performance. The current study extends these findings by showing that a comprehensive active learning approach grounded in the Knowledge Flow framework (Clabough, 2025) can achieve similar positive outcomes. While Clabough (2025) demonstrated that the Knowledge Flow redesign of this course significantly improved exam performance, the present study complements those findings by showing that the redesign also produced measurable improvements in students’ perceived learning experience and engagement as captured through AI-based text analysis of open-ended feedback.

### What the Discrepancy Between NLP and Likert-Scale Results Reveals

The comparison between our NLP results and the Likert-scale metrics revealed intriguing discrepancies. The Likert-scale means for enthusiasm and understanding were nearly identical across formats (3.68 vs. 3.81; 4.12 vs. 4.06), whereas the NLP analysis detected significant differences in learning experience and engagement. This pattern illustrates how aggregate Likert means can mask meaningful variation that open-ended responses reveal.

This discrepancy is especially important for neuroscience programs conducting course review or program assessment. If departments rely exclusively on standard numerical ratings to evaluate course effectiveness, they may be systematically underestimating the impact of pedagogical innovations. Incorporating open-ended evaluation analysis, even using the simplified workflow described here, into regular program assessment could provide a more accurate picture of teaching effectiveness and student experience.

Moreover, the zero-shot classification approach offers the potential for standardization across different courses and institutions. By using predefined categories, it allows for comparison across diverse educational contexts, facilitating more robust cross-institutional research on active learning effectiveness. This standardization is particularly valuable given the challenges of assessing active learning impacts in large enrollment courses, as highlighted by Knudson and Wallace (2021). The multi-dimensional analysis enabled by the LLM approach aligns with Carr et al.’s (2015) call for comprehensive measures to evaluate active learning effectiveness. The practical advantages of this approach, including open-source access, speed, and no requirement for labeled training data, are described in detail in Teles and Tomasevic (2026).

### Limitations

While the study offers valuable insights, it is important to acknowledge its limitations. First, we focused on student perceptions rather than direct measures of learning outcomes. Course grades were not included because the transition to active learning also involved changes to assignment structure and assessment design, making cross-semester grade comparisons invalid as a measure of instructional format. Future studies could address this limitation by incorporating standardized assessments administered consistently across both formats, such as concept inventories or common exam items, to correlate perceived and actual learning gains.

Second, individual-level validation of the LLM categories could not be conducted for the neuroscience course because the institutional evaluation system did not provide individual Likert-scale responses. The same classification method was validated on a separate course with positive evidence of discriminant validity (Teles & Tomasevic, 2026), but course-specific validation remains an important goal. Future studies should seek to obtain individual-level evaluation data to enable this.

Third, while advanced, LLM models can still misinterpret context or nuance in student responses. For example, in our dataset, a student comment describing appreciation for “being able to watch lectures at my own pace” received a moderately high Engaged More score, though the comment referred to the asynchronous lecture component rather than active participation in group sessions. Beyond misclassification, LLMs may also reflect biases present in their training data, including biases related to language style, cultural expression, or demographic characteristics of the writers (Bender et al., 2021). This means that classification scores may not be equally reliable across all student populations, and that systematic patterns in whose feedback is accurately captured may go undetected without targeted auditing. Educators wishing to adopt this approach are encouraged to implement a straightforward quality-checking workflow: after running the classification, randomly sample 10–15% of comments and manually verify whether the model’s assigned category aligns with a human reading of the text. Flagging comments where the model score seems inconsistent with the content, particularly those receiving high scores on categories that appear mismatched, can help identify systematic errors. Where misclassifications cluster around a particular type of comment (e.g., references to course logistics being coded as engagement), researchers may consider refining category label wording or excluding ambiguous prompt responses from the analysis. Combining this periodic human review with transparency about model limitations when reporting results will help ensure that AI-assisted feedback analysis is used responsibly and interpreted appropriately.

Finally, although a companion study applying the same assessment approach to an Introduction to Cognition course found comparable patterns (Teles & Tomasevic, 2026), suggesting the results may generalize, replication across diverse courses and institutions remains important.

### Practical Guidance for Neuroscience Educators

For neuroscience educators, these findings carry several practical implications. First, the pattern of improvement in perceived learning experience and engagement suggests that a hybrid active learning redesign can be meaningfully effective even without dramatic changes to course content: the same core topics and instructor can produce substantially different student outcomes through structural changes to how students engage with material. This is encouraging for instructors who may feel constrained by curriculum requirements or department norms.

The active learning design used here, grounded in cognitive science principles of retrieval and elaboration, aligns naturally with the content of neuroscience courses. Students in neuroscience courses are, in a sense, learning about the very mechanisms that underlie effective learning. Connecting the structure of the course to its content (for example, explicitly framing retrieval practice activities in terms of long-term potentiation or memory consolidation) may offer a unique pedagogical opportunity that other disciplines do not share. The Knowledge Flow framework (Clabough, 2025) makes this connection explicit, offering neuroscience educators a theoretically coherent rationale for their course design choices.

The course redesign described here is adaptable to a range of large-enrollment neuroscience courses. The hybrid format (asynchronous online lectures combined with weekly in-person active learning sessions) requires early-semester communication with students about the rationale and expectations for both components. Students initially expressed uncertainty about how to prepare for the in-person sessions; providing a one-page “session preparation guide” in the first week substantially reduced this confusion.

The full course enrollment of approximately 200 students was divided into two parallel active learning sections of 100 students each, covering identical content. Each section met in a 100-seat classroom configured with 11 tables of 9 to 10 students, enabling small-group work within what remained a large-enrollment course. Instructors without dedicated active learning spaces can approximate this structure by grouping adjacent rows of a lecture hall. The key design principle is ensuring that every activity requires students to produce something (a diagram, a short explanation, a prediction) that can be briefly shared or reviewed.

While this study focused on end-of-semester evaluations, the efficiency of LLM-based analysis suggests potential for more frequent, even real-time, assessment of student experiences. This could enable instructors to make data-driven adjustments to their teaching strategies throughout the semester, addressing the need for adaptive teaching methods in large classes as discussed by Schmidt and Libre (2020). To replicate the assessment approach used in this study, instructors can use the open-source R package *transforEmotion* (Tomasevic et al., 2022) to classify open-ended evaluation comments. A step-by-step guide with annotated code is available in Teles and Tomasevic (2026). For the current analysis, the full analysis of 547 comments ran in under two minutes on a standard laptop and requires no programming background beyond basic R familiarity.

## Conclusion

This study demonstrated the significant positive impact of active learning strategies in a large enrollment neuroscience course, as well as the effectiveness and unique value of advanced LLM techniques in analyzing student feedback. The discrepancies observed between the students’ comments and Likert-scale results in course evaluations highlight the importance of using diverse assessment methods to capture the full complexity of student experiences in evolving educational environments. As we continue to innovate in higher education pedagogy, the development of sophisticated, multi-faceted assessment tools will be crucial in understanding and optimizing the impact of these innovations on student learning and engagement. We invite neuroscience educators to adapt the active learning framework and assessment workflow described here for their own courses. All code, materials and detailed implementation guidance for the LLM analysis is available in Teles and Tomasevic (2026). Sharing replications, adaptations, and results, including null or mixed findings, across the neuroscience education community will be essential for building evidence strong enough to guide curriculum decisions in our field.

### Address correspondence to:

Mariana Teles (mt2yq@virginia.edu)

### Disclosure of Interest

The authors report no conflict of interest.
